# Gestational Exposure to the Synthetic Cathinone Methylenedioxypyrovalerone Results in Reduced Maternal Care and Behavioral Alterations in Mouse Pups

**DOI:** 10.3389/fnins.2018.00027

**Published:** 2018-02-05

**Authors:** László I. Gerecsei, András Csillag, Gergely Zachar, Lőrinc Gévai, László Simon, Árpád Dobolyi, Ágota Ádám

**Affiliations:** ^1^Department of Anatomy, Histology and Embryology, Semmelweis University, Budapest, Hungary; ^2^Laboratory of Sensorimotor Adaptation, Semmelweis University, Budapest, Hungary; ^3^MTA-ELTE Laboratory of Molecular and Systems Neurobiology, Institute of Biology, Hungarian Academy of Sciences and Eötvös Loránd University, Budapest, Hungary

**Keywords:** new psychoactive substances, synthetic cathinone, bath salt, maternal care, methylenedioxypyrovalerone, locomotor activity, pregnancy

## Abstract

The member of synthetic cathinone family, methylenedioxypyrovalerone (MDPV), is a frequently used psychoactive drug of abuse. The objective of our study was to determine the effect of MDPV (administered from the 8th to the 14th day of gestation) on the behavior of neonatal and adolescent mice, as well as its effect on maternal care. We measured maternal care (pup retrieval test, nest building), locomotor activity (open field test), and motor coordination (grip strength test) of dams, whereas on pups we examined locomotor activity at postnatal day 7 and day 21 (open field test) and motor coordination on day 21 (grip strength test). On fresh-frozen brain samples of the dams we examined the expression of two important peptides implicated in the regulation of maternal behavior and lactation: tuberoinfundibular peptide 39 (TIP39) mRNA in the thalamic posterior intralaminar complex, and amylin mRNA in the medial preoptic nucleus. We detected decreased birth rate and survival of offspring, and reduced maternal care in the drug-treated animals, whereas there was no difference between the motility of treated and control mothers. Locomotor activity of the pups was increased in the MDPV treated group both at 7 and 21 days of age, while motor coordination was unaffected by MDPV treatment. TIP39 and amylin were detected in their typical location but failed to show a significant difference of expression between the drug-treated and control groups. The results suggest that chronic systemic administration of the cathinone agent MDPV to pregnant mice can reduce birth rate and maternal care, and it also enhances motility (without impairment of motor coordination) of the offspring.

## Introduction

Synthetic cathinones, which are structurally similar to methcathinone, an extract of khat plant (*Catha edulis*) (Magdum, 2011) represent an important class of new psychoactive substances (NPS). In 2015, synthetic cathinones were the most commonly confiscated group of NPS in the European Union and their percentage is rising (EMCDDA, 2017). Synthetic cathinones are mainly used for recreational purposes, as inexpensive replacements for other illicit stimulant drugs (Prosser and Nelson, 2012). Widespread use of NPS is strongly assisted by the anonymous services of the Internet (e.g., dark web sites, the Tor search engine, Bitcoin, and other crypto currencies) and the belief in legality by the potential users (Regan et al., 2011; EMCDDA, 2017). It is important to mention that the legal status of the specific NPS may or may not significantly influence their usage (Dybdal-Hargreaves et al., 2013; Kriikku et al., 2015; Sande, 2016), therefore, both the recent and the old (better known) substances can remain on the market simultaneously. However, marked geographical differences can be observed even within Europe (for country specific reports see: Szily and Bitter, 2013; Kriikku et al., 2015; Adamowicz et al., 2016; Odoardi et al., 2016; Sande, 2016; Romanek et al., 2017). Although most reports on the prevalence of NPS usage state that the majority of the users are males (Beck et al., 2017; Romanek et al., 2017), there have been case reports on synthetic cathinone abuse among women in pregnancy (Gray and Holland, 2014; Pichini et al., 2014). NPS are generally used by socially marginalized and otherwise high-risk groups during pregnancy (Minnes et al., 2011; EMCDDA, 2017).

Abuse of synthetic cathinones caused an emerging health concern throughout the U.S and Europe (Carroll et al., 2012; Glennon, 2014). The primary effects sought by users include increased alertness, empathy, euphoria, talkativeness, intensification of sensory experiences, reduced appetite, insomnia, enhanced sexual performance, increased sociability, and capacity to work (Karila and Reynaud, 2011; German et al., 2014). Apart from “desired” effects, however, a number of adverse, sometimes health-threatening effects have also been reported by users and clinicians: agitation, combative behavior and tachycardia in most users, while many suffered from hallucinations, psychosis, paranoia, confusion, chest pain, myoclonus, and hypertension after consumption of 3,4-methylenedioxypyrovalerone (MDPV) (Spiller et al., 2011; Farkas et al., 2013; Beck et al., 2015; White, 2016), and other synthetic cathinones (Karila et al., 2015; Vallersnes et al., 2016; Romanek et al., 2017).

MDPV is an important member of the synthetic cathinone family (Spiller et al., 2011), which acts as a monoamine transport inhibitor, with primary actions on dopamine and norepinephrine transporters, while having minimal effect on serotonin transport (Baumann et al., 2013; Simmler et al., 2013; Rickli et al., 2015; Shekar et al., 2017). MDPV is self-administered by laboratory rodents (Aarde et al., 2013, 2015; Bonano et al., 2014; Watterson et al., 2014) and is known to induce conditioned place preference (Karlsson et al., 2014; King et al., 2015). Acute injection of MDPV increases locomotor activity both in rats (Gatch et al., 2013) and in mice (Fantegrossi et al., 2013; Gannon et al., 2017), and it also enhances stress-related behavior and wakeful activity in day-old domestic chicks (Zsedényi et al., 2014). Although more and more information is being gathered about MDPV and other frequently used synthetic cathinones, apart from the above mentioned case reports (Gray and Holland, 2014; Pichini et al., 2014), the literature still lacks data about the effect of these substances on maternal care, maternal behavior as well as on newborn pups/babies. Investigation into these questions would be important not just because women in childbearing age can be found among the users of synthetic cathinones (see above) but some relevant data also suggest potential negative consequences of synthetic cathinone abuse during pregnancy. Khat chewing, which is common in the Middle-East, correlates with low birth weight and intrauterine growth restriction (Hassan et al., 2007; Abdel-Aleem et al., 2015), however these studies can barely separate their results from the khat-chewers' other severe socio-demographical risk factors. 3,4-Methylenedioxymethamphetamine (MDMA) exposure also has known adverse effects on the developing brain and behavior (Skelton et al., 2008). A recent study showed that in mice MDPV crosses the placental barrier and reaches higher concentration in fetal brain than the structurally similar mephedrone and methylone. In addition, MDPV has longer half-life *in utero*, which means prolonged drug exposure for the fetal brain (Strange et al., 2017). Even a single injection of 10 mg/kg of MDPV to 7-day-old mouse pups was followed 24 h later by neurodegeneration (enhanced apoptosis) in specific brain regions, including the striatum and nucleus accumbens (Ádám et al., 2014). This marked morphological alteration, not reproducible in adult mice, was accompanied by increased locomotion, as detected by open field test.

Selective sensitivity of young developing individuals to cathinone-related neurodegeneration raised the possibility of a direct impairment of pregnant mothers and their offspring *in utero*. To this end, MDPV was administered to pregnant mouse mothers on a daily basis, in a critical phase of ontogeny. Pups were tested for body weight, motor activity and coordination at 7 and 21 days of age, while the dams were tested for any deficiencies in maternal care. The latter investigation included behavioral tests (prenatal nest building and postnatal pup retrieval), as well as brain expression of two neuropeptides relevant for maternal adaptation: amylin and tuberoinfundibular peptide of 39 (TIP39) (Dobolyi, 2011).

TIP39 and amylin genes show a high induction between the *prepartum* and the *postpartum* periods (Dobolyi, 2011). Their mRNA expression is low during pregnancy, while it is high on the 1st *postpartum* day and, later on, during lactation. The expression of amylin mRNA in the medial preoptic area correlates with maternal behavior and is induced by the physical presence of pups, most importantly by suckling (Szabó et al., 2012). The expression of TIP39 mRNA in the posterior intralaminar complex of the thalamus (PIL) is also correlated with maternal behavior (Dobolyi, 2011; Cservenák et al., 2013). The TIP39 neurons of the PIL region constitute a relay nucleus that conveys the somatosensory afferents due to pup exposure, which is most important for sustained maternal behavior (Cservenák et al., 2013; Scott et al., 2015). Based on a plethora of experimental data and correlative functional evidence, we consider amylin and TIP39 to be relevant neural correlates of maternal adaptation.

Overall, we hypothesized that, if the synthetic cathinone MDPV were administered chronically during pregnancy to mouse dams, it might have an effect on the behavior of the offspring (more precisely on locomotor activity and neuromuscular control), as well as on maternal behavior and maternal care. We also assumed that, given an impairment of the maternal behavior of MDPV-treated dams, it could be evaluated by the detection of TIP39 and amylin mRNA in relevant brain areas, for better understanding of the mechanisms underlying deficits of pre- and post-natal maternal behaviors and maternal care.

## Materials and methods

### Animals and drugs

C57Bl/6J mice were mated in the animal facility of the Semmelweis University, Department of Anatomy. All pregnant animals were first-time dams, their age ranging between 16 and 22 weeks. Vaginal plugs were checked in each case 12–24 h after mating. Pregnant females were housed individually under standard laboratory conditions (12 h dark/12 h light cycle) with food and water available *ad libitum*. The progress of pregnancy was monitored by daily measurement of the gain of body weight. Pregnant females (*n* = 40) were randomly assigned to either the experimental or the control group. The experimental group received 10 mg/kg MDPV in 0.9% sterile saline, by subcutaneous injection in the suboccipital region, whereas the control group received an equivalent volume of 0.9% sterile saline. At first sight, the 10 mg/kg dose of MDPV might be considered rather high, however, it has been used by many authors investigating the behavioral effects of MDPV, such as conditioned place preference (Karlsson et al., 2014), locomotor stimulation (Marusich et al., 2012; Gatch et al., 2013; Gannon et al., 2017) and neuromuscular control (Marusich et al., 2012), without reporting lethality or severe acute side effects. In addition, our own preliminary results in MDPV-treated adult mice showed no severe side effects or lethality, either, while visible alterations of behavior (increased locomotor activity, stereotypies) were clearly detectable. Using the same dose, we have also observed increased locomotor activity and apoptotic cell death in several brain regions in neonatal mice (Ádám et al., 2014). In the present study, the drug was administered daily over a 7-day gestational period, from E8 to E14, which mirrors the early appearance of dopaminergic neurons and the mesolimbic system. The experiments were conducted in conformity with the laws and regulations controlling experiments and procedures in live animals, as described in the Principles of Laboratory Animal Care (NIH Publication 85-23, revised 1985). Our Department is in possession of a valid experimental license for working with laboratory animals including mice, issued by the Food Chain Safety and Animal Health Directorate of the Government Office for Pest County, Hungary (license number: XIV-I-001-2269-4/2012).

The racemic mix of MDPV was produced by LGC Standards (Teddington, Middlesex, UK) and purchased through Medinspect Kft (Fót, Hungary). The experiments were carried out under a special license (#27924/2011/KÁB) issued by the Narcotic Drugs Control Department, Office of Health Authorization and Administrative Procedures, Hungary.

### Open field test

Locomotor activity was evaluated by placing the mice into the center of an open field arena (30 × 30 × 30 cm) for 10 min. We recorded the number of times the mouse crossed a grid line of the 3 cm by 3 cm rectangular array drawn on the floor of the arena. Notably, this method of testing also carries an element of novelty-induced activity measurement. The test was run on the pups of the chronically MDPV-treated mothers at two different ages: day 7 (*n* = 14 for the MDPV-treated group and *n* = 15 for the saline group) and day 21 (*n* = 14 for the MDPV-treated group and *n* = 14 for the control group). It has to be noted that 7-day-old mice pups are blind, a hindrance to normal exploratory behavior as observed in adults, even if laboratory rodents are known to rely on tactile stimuli, too.

### Grip strength test

Grip strength test is often used to assess neuromuscular and locomotor development (Crabbe et al., 2003). In the present study, two groups of animals were tested: 21-day-old offspring of injected mothers (*n* = 14 for the MDPV-treated group and *n* = 14 for the control group), as well as the dams 7 days after parturition (*n* = 6 for the MDPV group and *n* = 10 for the control group). The mice were placed on a horizontal non-rotating metal dowel of 3.5 mm diameter, spanning the distance between opposite sides of a square box (side length 25 cm) ca. 25 cm above ground level. The animals instinctively hang on to the metal rod using the front paws, until they lose grip and fall on the bottom of the box (this impact presents no harm to the animals). The latency to fall down was measured. This test was taken for a measure of locomotor control: the longer time they spend hanging onto the device, the better motor coordination they display. We tried to avoid any effect of locomotor (procedural) learning, therefore the animals were not tested repeatedly, the maximum number of tests per individual being two, at 30 min intervals. On the other hand, the animals were allowed a second trial if they fell immediately (within 1 s), on their first attempt, in order to eliminate the confounding effect of surprise by novelty.

### Force plate actometry

The recording system was developed by the Laboratory of Sensorimotor Adaptation. The principle of recording, first adapted to human posturography for monitoring of positioning maneuvers of subjects, has been reported previously (Büki et al., 2011). The apparatus in the present form was recently employed also in small animal (mouse) experiments (Szabó et al., 2015). The system records the motor responses of the laboratory animal placed on a metal platform, which is bolted to four linear load cells as force transducers in a quadrangular array. The force transducers at each corner record the vertical load, and the center of gravity (COG) is computed at a frequency of 108 CPS by the recording algorithm. The signal is digitized onto an X-Y grid, with an optimized spatial resolution, yielding a track record of the sway of COG in the form of a coil (tangle). The total measuring field (300 × 120 mm) extended to 96 quadrangular sectors. For a typical recording, the apparatus was placed on a solid surface in a quiet experimental room with low noise and vibration. To prevent shivering artifacts, the animals were kept warm by an infrared lamp (150 W), placed over the platform ~60 cm apart. Prior to measurements, the system was tested “blank” by placing a 2 × 3 cm cylindrical metal object in the middle of the platform. Animal testing was commenced only if the recording track in the blank test remained within the confines of one sector. Blank tests were repeated every time before starting a new animal recording. The animals were placed in the center of the platform and allowed to move about freely for 5 min (*n* = 14 for the MDPV-treated group and *n* = 15 for the saline group). The parameters calculated were the distance traveled (a typical metric for locomotion) and the number of sectors occupied (a measure for areal coverage). Although the measuring algorithm had been optimized for accentuating locomotion and reducing contribution of other oscillatory movements to the distance variable, the final score does take into account the behavior of mice even when they stayed in one place. Non-ambulatory movements (head, limbs, trunk), if sufficiently vigorous, also contributed to deflection of the COG. While we did not intend to calculate focused stereotypy scores, used mainly for adult animals of wider behavioral repertoire, force plate actometry here was considered a useful tool for the testing of pups of this age (lacking vision and with highly erratic motor capabilities), altogether yielding a more complex metric of locomotion than what could be deduced from open field tests alone.

### Pup retrieval test

Pup retrieval is a well-established test to measure maternal motivation and behavior (Porter, 1983). The test was performed in the home cage on postnatal day (PD) 7 (*n* = 6 for the MDPV-treated group, and *n* = 7 for the control group). After removing the dam from the cage, pups were placed in the farthest corner away from the nest (~30 cm away). The dam was then returned to the original nest position. The time elapsed between reintroduction of the dam and touching the first pup, as well as the latency to retrieve all the pups to nest position, were recorded. There was a time ceiling at 600 s if the dam failed to retrieve her pups.

### Nest building of pregnant mothers

Nests built by the pregnant females were rated at two time points: at day 6 of pregnancy (i.e., prior to drug injections), and again at day 12 (i.e., well into chronic drug treatment but with sustained pregnancy). We provided the animals with equal amount (2.5 g each) of pressed cotton nesting material for small rodents (commercially available in pet shops), placing a pile of material at the top of the cage from where the animals could pull the strands of cotton fiber in. For measuring the quality of the nest we used a definitive 5-point nest-rating scale, as described by Hess et al. (2008). Scores are based on the shape of the nest as well as to what degree the walls are built up around the nest cavity in order to form a dome. In particular, mice were counted as having built a nest only if the nesting material contained a central hollow. Nests were then further scored according to the height and closure of the walls surrounding the nest cavity. The score indicated the quality of the nest: higher scores indicated higher quality nests, whereas those with lower scores were of poor quality.

### *In situ* hybridization

*In situ* hybridization probes were generated as described previously (Dobolyi et al., 2006; Cservenák et al., 2010; Szabó et al., 2012). Briefly, total RNA was isolated from freshly dissected diencephalon. The concentration of the RNA was adjusted to 2 μg/μL before it was treated with Amplification Grade DNase I (Invitrogen). The cDNA was synthesized using SuperscriptII (Invitrogen) and after a 10-fold dilution, 2.5 μL of the resulting cDNA was used as a template in PCR reactions. The primers (Table 1) were used at 300 nM concentration. The PCR reaction was performed using iTaq DNA polymerase (Bio-Rad Laboratories, Hercules, CA). The PCR product was purified from the gel, inserted into TOPO TA cloning vectors (Life Technologies) and transformed chemically into competent bacteria. Selected plasmids were used as templates in PCR reactions, using the primer pair specific for TIP39, with the reverse primers also containing a T7 RNA polymerase recognition site. Finally, the identity of the cDNA probe was verified by sequencing (Biomi Ltd, Gödöllő, Hungary).

**Table 1:** Accession numbers and corresponding PCR primers used in the present study.

**Name of peptide**	**Short gene name**	**UniGene code**	**GenBank accession number**	**Sequence of primers**	**Position of primer sequence**
Tuberoinfundibular peptide 39 (TIP39), or Parathyroid hormone 2	Pth2	Mm. 207078	NM_053256	Forward: CTGCCTCAGGTGTTGCCCT	289–307
				Reverse: TGTAAGAGTCCAGCCAGCGG	452–471
Amylin, or Islet amyloid polypeptide	Iapp	Mm. 415	NM_010491	Forward: CTCTCTGTGGCACTGAACCA	138–157
				Reverse: TTCAGGAAATCACCAGAGCA	422–441

To investigate the expression level of TIP39 in the PIL region, brains of 4 MDPV-treated and 3 saline-treated mothers were removed 10 days *post partum*, the tissue was quickly frozen on dry ice and stored at −80°C until use. *In situ* hybridization histochemistry was carried out as described previously (Dobolyi et al., 2002). Briefly, 12-μm-thick coronal sections were cut with a cryostat at +0.5 to −0.5 mm from the bregma level, mounted immediately onto positively charged slides (SuperfrostUltraPlus, Thermo Fischer Scientific, Pittsburgh, PA), dried and stored at −80°C. Antisense [^35^S]UTP-labeled riboprobes were generated using T7 RNA polymerase of the MAXIscript transcription kit (Ambion, Austin, TX). Eighty microliters of hybridization buffer and 106 DPM of labeled probe per slide were used for hybridization. Every 9th coronal section was hybridized to visualize the TIP39 mRNA expression at 108 μm distances. The washing procedure included a 30 min incubation in RNase A, followed by decreasing concentrations of sodium-citrate buffer at room temperature, and then at 65°C. After drying, the slides were dipped in NTB nuclear track emulsion (Eastman Kodak, Rochester, NY) and stored at 4°C for 3 weeks for autoradiography. Then, the exposed slides were developed and fixed with Kodak Dektol developer and Kodak fixer, counterstained with Giemsa, dehydrated, and finally coverslipped.

For amylin, *in situ* hybridization was performed as described above, except that the coronal sections were cut at −2.8 to −3.5 mm from bregma level, corresponding to the location of medial preoptic nucleus (MPO). We used the same animals for these experiments (4 MDPV-treated subjects and 3 controls) as for the TIP39 experiments.

Both for TIP39 and amylin, mRNA-containing neurons were counted manually on micrographs taken from 3 consecutive coronal sections (Olympus BX 50 microscope equipped with a digital camera, 20x objective lens), in which the TIP39 or amylin signals were found to be most intense in the PIL or MPO, respectively. Silver grain density over TIP39 or amylin expressing neurons was calculated in each series of brain sections, using ImageJ software (NIH, USA). The resulting figures were normalized for background by dividing these by the grain density values obtained from a frame of equal size on the same section, which contained only background grains. The ratio of these values was used to compare the TIP39 and amylin mRNA expression levels of the treatment groups.

### Statistical evaluation

The open field test and grip test results of 7 and 21-day-old offspring were evaluated by using multivariate ANOVA with the test results as variables, the maternal drug treatment as fixed factor and the litter as covariate. Mann-Whitney *U*-test (M-W. *U*-test) was used to analyze pup retrieval: the latency to first touch and the time required for returning all pups to the nest (divided by the number of pups in litter). The *t*-test was used to analyze the results of force plate actometry and body weight in all groups. Welch's version of *t*-tests was applied in those cases where the variances differed among the groups compared. For the analysis of *in situ* hybridization signals, Student's *t*-test was performed, using Statistica v.12 software.

## Results

### Number and survival of pups, body weight

A significant decrease of survival rate was observed among the offspring of MDPV-treated mothers, the mean pup/litter number at day 7 being 4.43 ± 1.1 (MDPV) and 6.30 ± 0.40 (control), *p* < 0.05. Whilst all vehicle-treated (control) pregnant females gave live birth at due time, the birth rate of MDPV treated animals was markedly smaller, with further reduction of pup survival due to stillbirth, premature birth, or cannibalism of dams (Table 2).

**Table 2:** Birth statistics of pregnant dams.

	**Nr. of live birth**	**Nr. of premature birth**	**Nr. of miscarriage**	**Nr. of stillbirth**	**Nr. of cannibalism**
MDPV-treated	18	2	18	2	4
Vehicle-treated	32	–	–	–	–

*The numbers represent pregnant dams at which the above categories of events occurred*.

**Figure 1:**
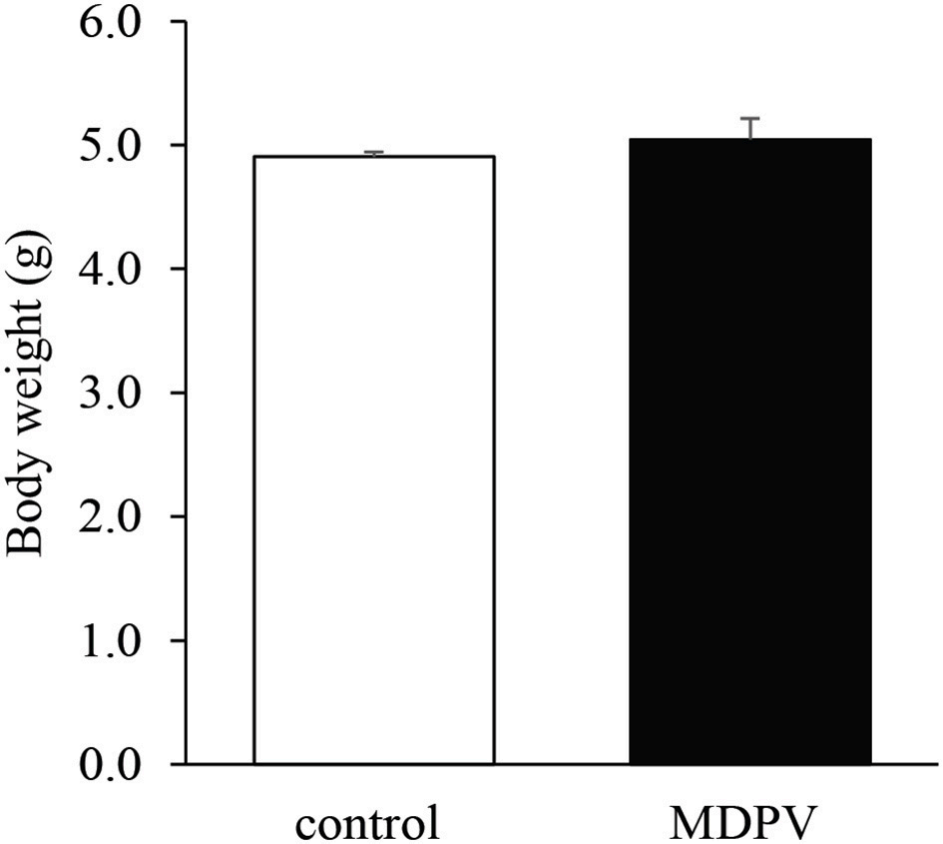
Effect of prenatal MDPV (10 mg/kg) treatment on body weight in 7-day-old mouse pups following s.c. administration to the mother, between gestational days 8 and 14. There was no difference in the body weight of the pups. The columns indicate mean + S.E.M.

There was no difference in the mean body weight of the pups between the treatment groups (Welch's *t* = 0.78, d.f. = 27, *p* = 0.43; Figure 1).

### Open field test

**Figure 2:**
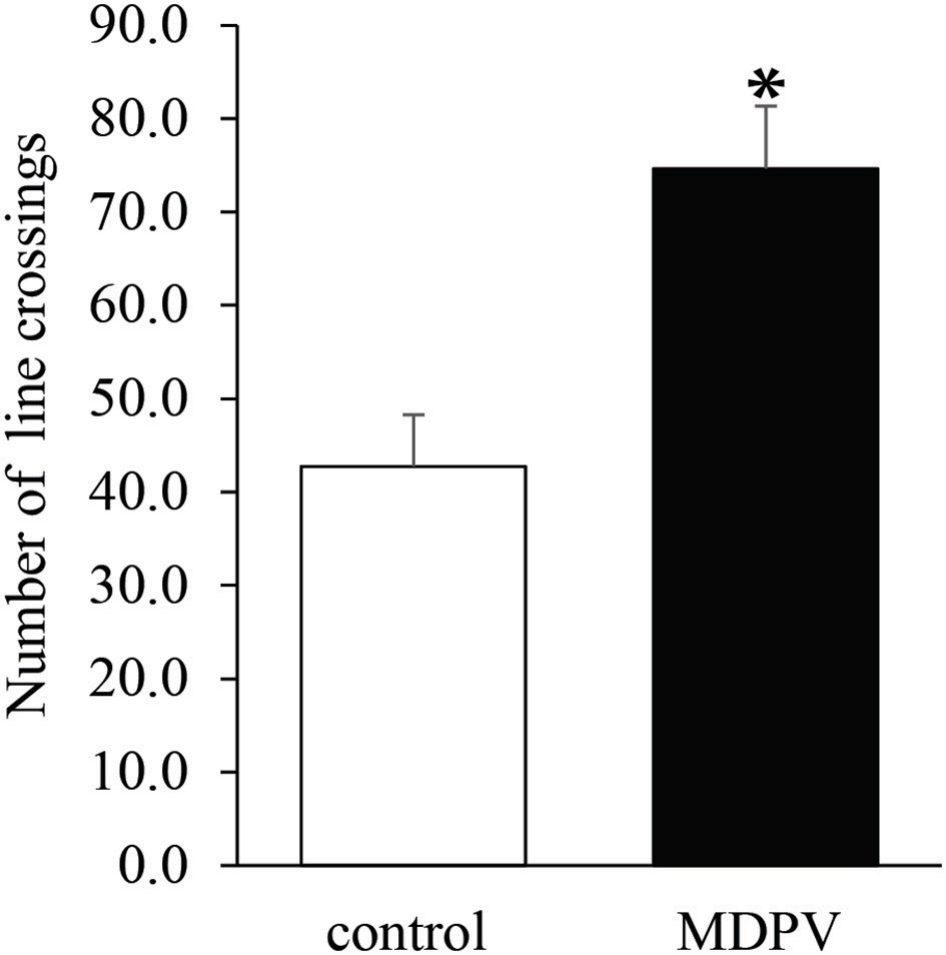
Effect of prenatal MDPV (10 mg/kg) treatment on locomotor activity in 7-day-old mouse pups following s.c. administration to the mother, between gestational days 8 and 14. The open field test revealed a significant increase in locomotor activity of MDPV-treated animals. The columns indicate mean + S.E.M. Significance level: ^*^p < 0.05.

**Figure 3:**
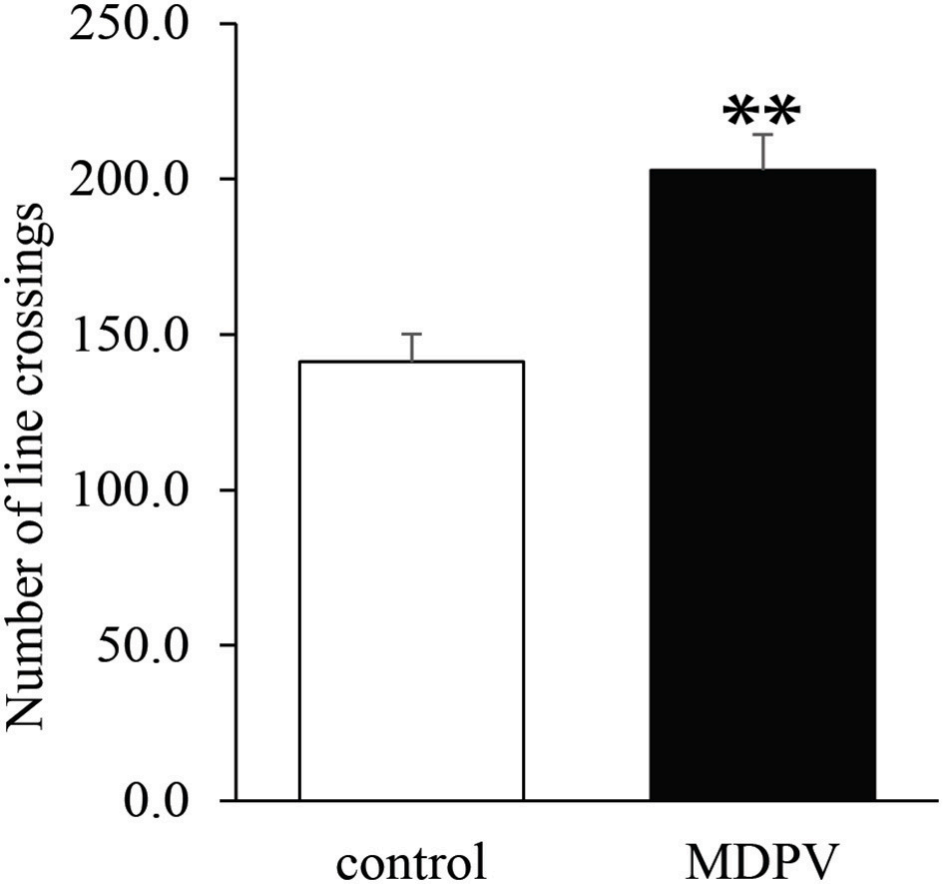
Effect of prenatal MDPV (10 mg/kg) treatment in open field activity of 21-day-old mouse pups following s.c. administration to the pregnant mothers, between gestational days 8 and 14. We found a significant increase in locomotor activity of pups. The columns indicate mean + S.E.M. Significance level: ^**^p < 0.001.

Both in 7-day-old and 21-day-old pups, the litter had no effect on the open field activity (ANOVA: *F* = 1.89, d.f.: 28, *p* = 0.181 and *F* = 0.1, d.f.: 26, *p* = 0.754, respectively), however, prenatal MDPV-treatment (Figure 2), significantly increased the locomotor activity of PD 7 pups (ANOVA: *F* = 9.5, d.f.: 28, *p* = 0.005). In the case of 21-day-old pups, there was a non-significant effect of the prenatal MDPV-treatment on the open field activity of 21-day-old offspring, too (ANOVA: *F* = 2.95, d.f.: 26, *p* = 0.099). If the covariate (litter) was omitted from the model, the prenatal treatment significantly increased the locomotor activity of the offspring (ANOVA: *F* = 18.2, d.f.: 26, *p* < 0.001) (Figure 3). However, the chronically treated mothers showed no difference in locomotor activity when compared to vehicle-treated control animals (Student's *t* = 2.94, d.f. = 10, *p* = 0.713).

### Grip strength test

**Figure 4:**
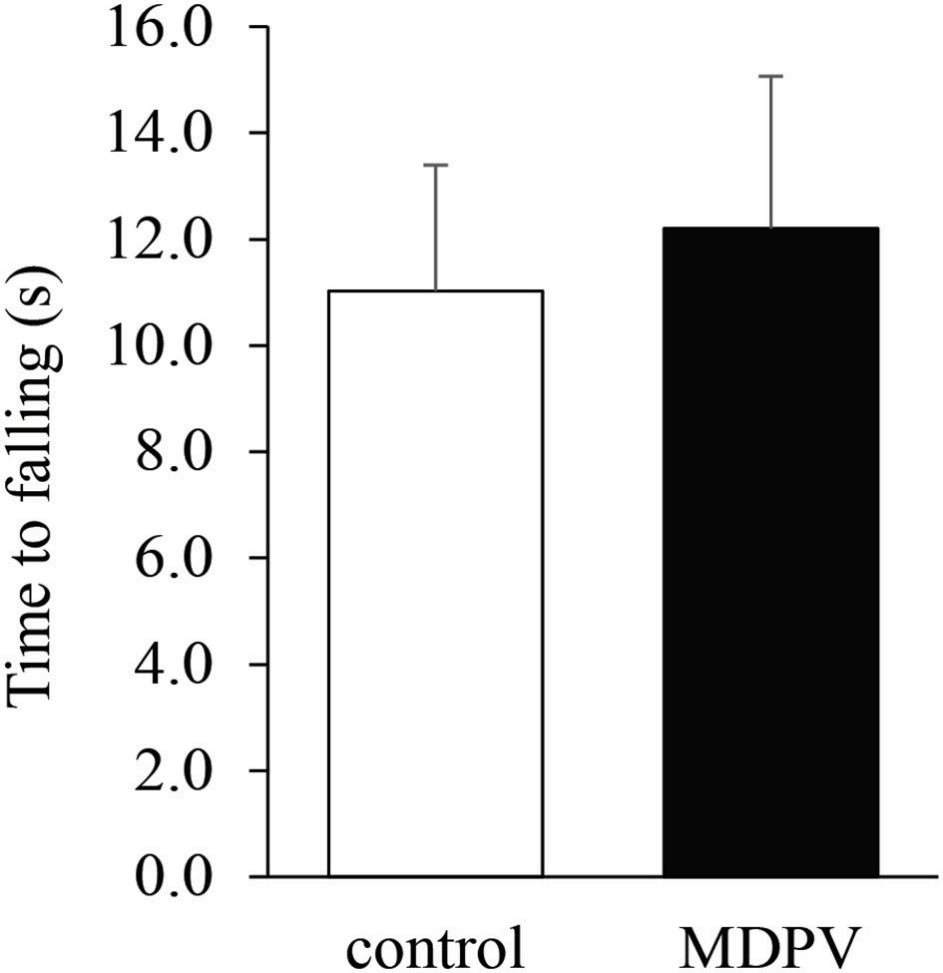
Effect of prenatal MDPV (10 mg/kg) treatment on grip strength test of 21-day-old mouse pups following s.c. administration to the pregnant mothers between gestational days 8 and 14. No difference in motor coordination of pups was found. The columns indicate mean + S.E.M.

**Figure 5:**
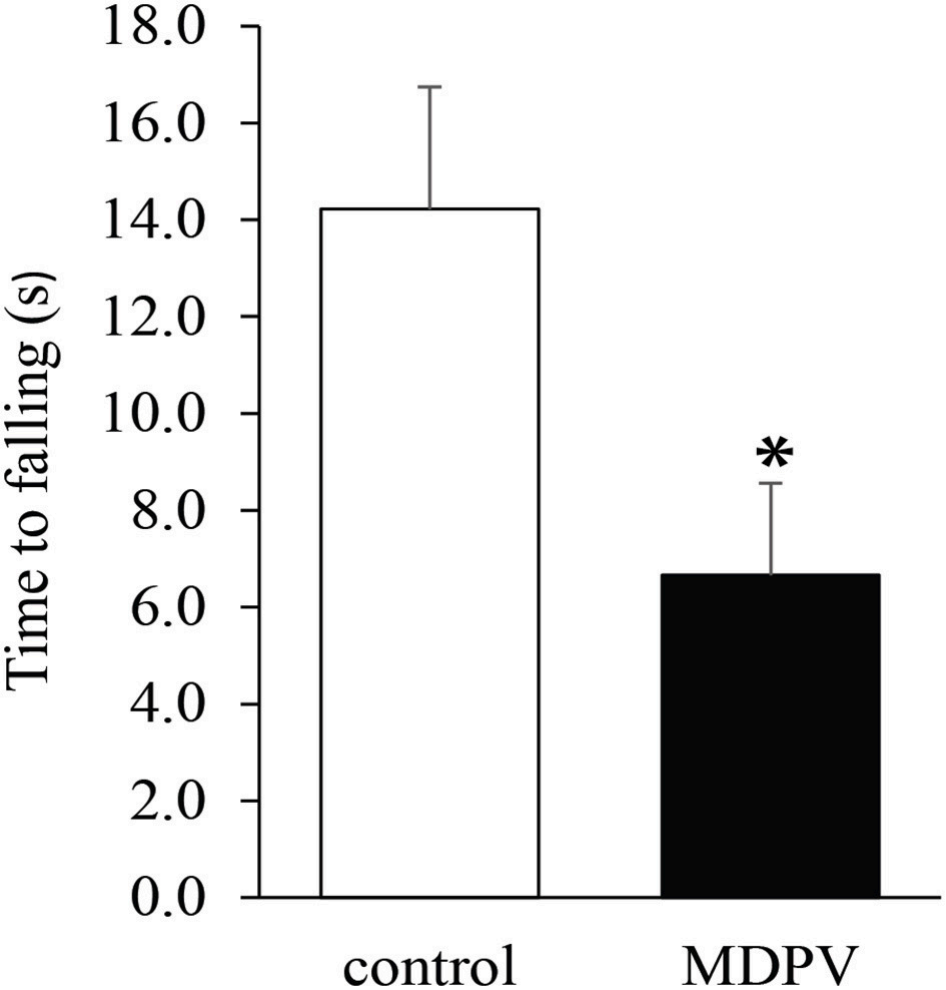
Effect of chronic exposure to MDPV (10 mg/kg for 7 consecutive days) on the grip strength test performance of adult female mice. The MDPV treated group had significantly diminished performance in grip strength test. The columns indicate mean + S.E.M. Significance level: ^*^p < 0.05.

Due to muscular weakness and insufficient postural coordination, this test cannot be performed on 7-day-old pups. However, it was feasible on the older postnatal group. There was no significant difference in the latency of losing grip between the MDPV-treated and control pups on postnatal day 21, after chronic administration of the drug during gestation (Figure 4; ANOVA: *F* = 0.3, d.f.: = 26, *p* = 0.596). Notably, the mothers, which had been exposed to chronic MDPV treatment alongside with their pups, showed a decreased latency of losing grip, as compared to control animals receiving vehicle instead of the drug (Student' *t* = 4.01, d.f. = 10, *p* = 0.002; Figure 5).

### Force plate actometry

**Figure 6:**
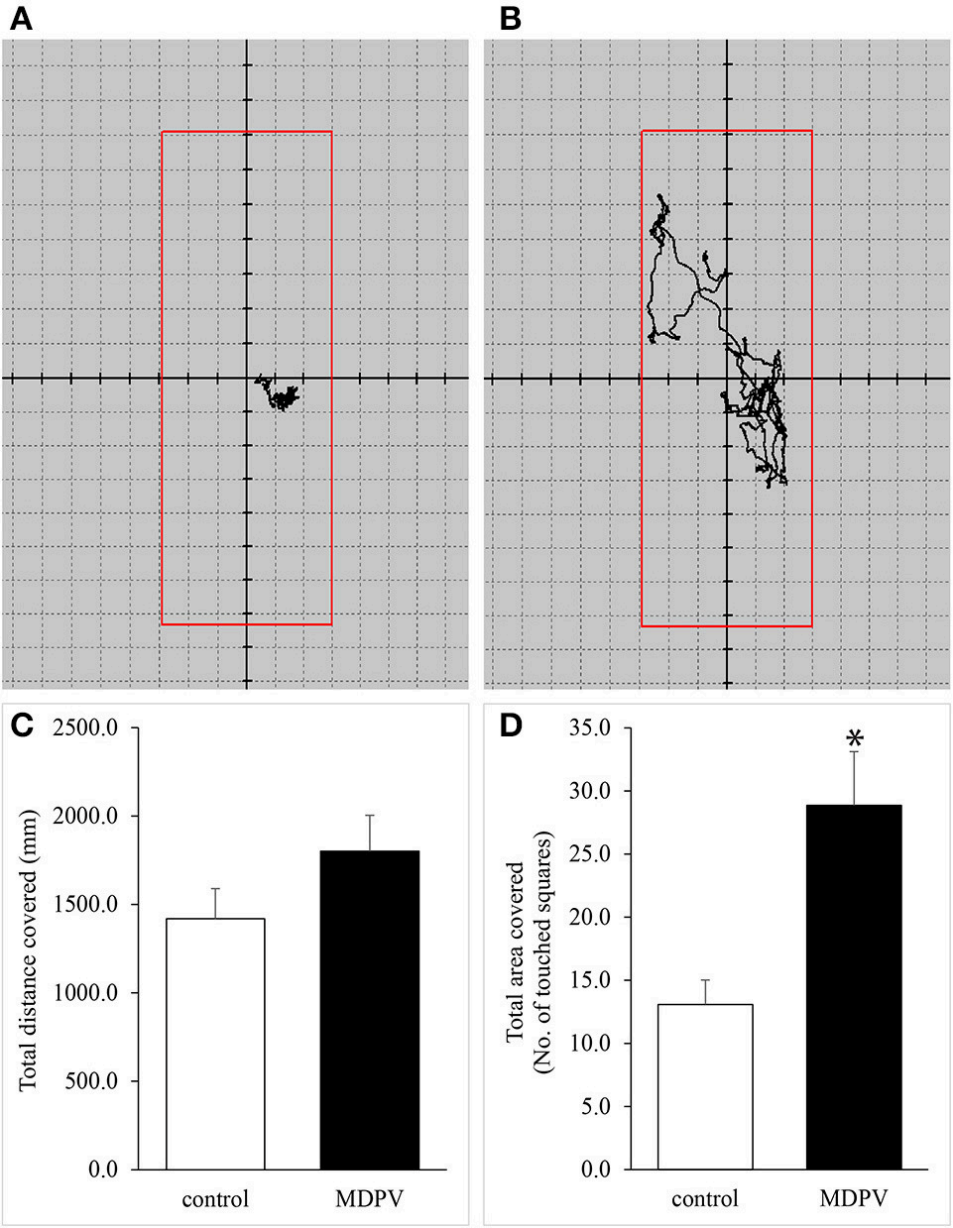
**(A,B)** Representative recordings of movement patterns of 7-day-old mouse pups detected with force plate actometry. There was a visible difference between the tracking “coils” of the control **(A)** and MDPV-treated **(B)** groups. **(C,D)** Effect of prenatal MDPV (10 mg/kg) treatment on motility of 7-day-old mouse pups, detected with force plate actometry. There was no difference in total distance traveled between the control and MDPV-treated groups **(C)**. We found a significant increase in the number of areal sectors (squares) occupied by the MDPV-treated group. The columns indicate mean + S.E.M (^*^p < 0.05) **(D)**.

Representative recordings are shown in Figures 6A,B, where the difference between the tracking “coils” can be observed. In spite of a distinct tendency of increased motility in the pups with chronic MDPV treatment, no significant difference in total distance traveled was found between the control and MDPV-treated groups (Figure 6C). However, there was a significant increase in the number of areal sectors occupied by the MDPV-treated group (Figure 6D), supporting the data obtained by open field tests.

### Maternal care (pup retrieval test)

**Figure 7:**
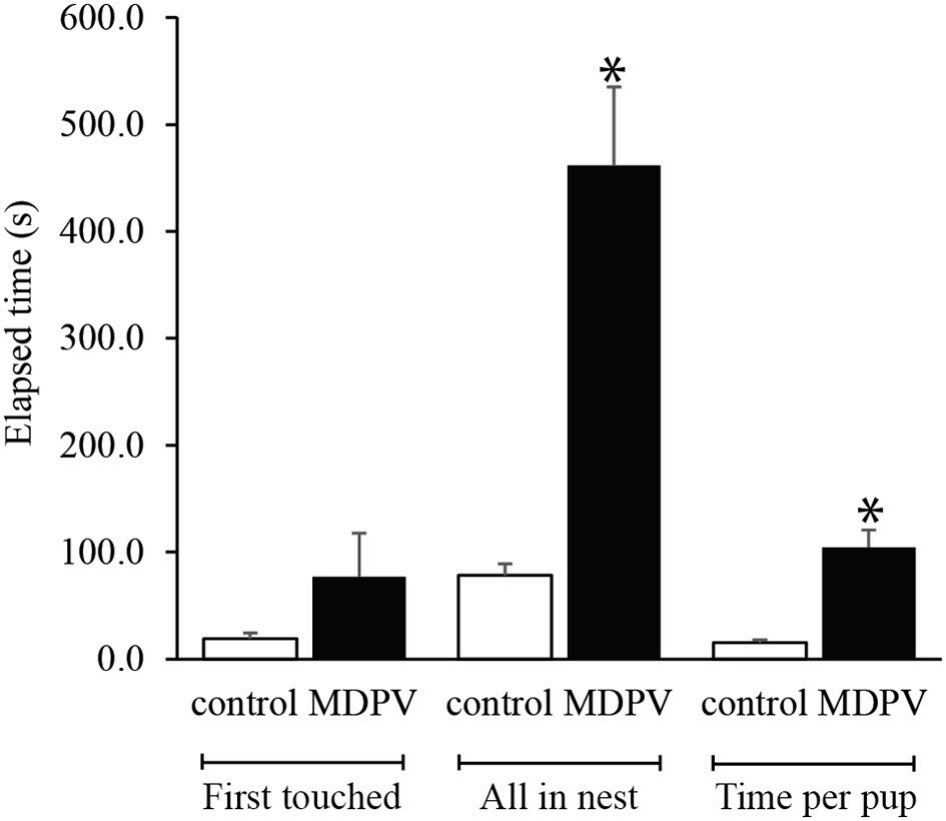
Effect of chronic MDPV-treatment during pregnancy on maternal care. Pregnant mice were treated s.c. with 10 mg/kg MDPV between gestational days 8–14 and were tested for pup retrieval on postnatal day 7. There was a significant increase in the latency to retrieve all pups to the nest (when standardized by the number of pups) (all in nest), and the average time required for returning one pup to the nest (time per pup) was longer in case of MDPV-treated animals. However, the first pup was touched at the same time point by the animals of the treatment groups (first touched) (^*^p < 0.05).

MDPV-treatment markedly altered the mothers' performance in the pup retrieval test. Although there was no difference between the elapsed times until the first pup was touched (M-W. *U*-test: *Z* = 0.968, *p* = 0.332), there was a significant increase in the latency to retrieve all pups to the nest (when standardized by the number of pups) (M-W. *U*-test: *Z* = 3.033, *p* = 0.0024). In addition, the average time spent on returning one pup to the nest was longer in case of MDPV treated dams (M-W. *U*-test: *Z* = 3.033, *p* = 0.0024; Figure 7).

### Nest building of pregnant mothers

**Figure 8:**
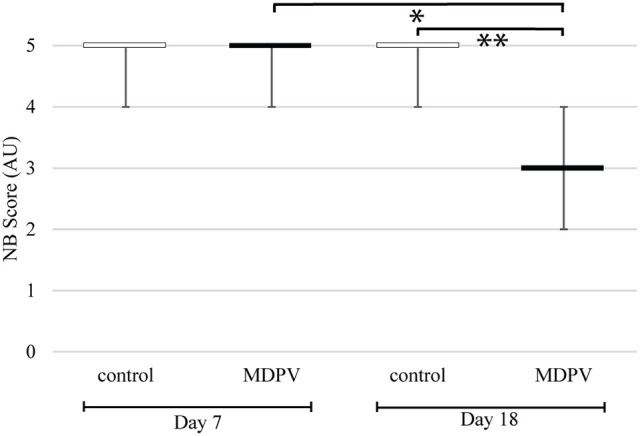
Nest building (NB) scores of pregnant dams. The scores are in arbitrary units (AU), estimated according to the criteria by Hess et al. (2008). Each box plot indicates the median, which in these cases equals to both the first and the third quartiles. The whiskers represent the minimum and maximum values. Significant difference between the MDPV-treated and the control dams was found on the 18th day of pregnancy (^**^p < 0.001). Comparing the same MDPV-treated individuals at day 7 and at day 18, the NB score of the latter group was significantly reduced (^*^p < 0.05).

Nest building (NB) scores of MDPV treated mothers did not differ from those of control mothers when rated on the 7th day of pregnancy (i.e., prior to the treatment). By contrast, the nest scores of drug treated mothers were significantly lower than those of control animals, when compared post-treatment, on day 18 of pregnancy (Figure 8) (M-W. *U*-test; on day 7: Z = −0.040, *p* = 0.967; on day 18: *Z* = −3.470, *p* = 0.000520). Comparing the same MDPV-treated individuals at day 7 and day 18, the NB score of the latter group was significantly reduced (Wilcoxon test: Z = 2.665, *p* = 0.00768).

### Expression of hypothalamic peptides (*in situ* hybridization histochemistry)

**Figure 9:**
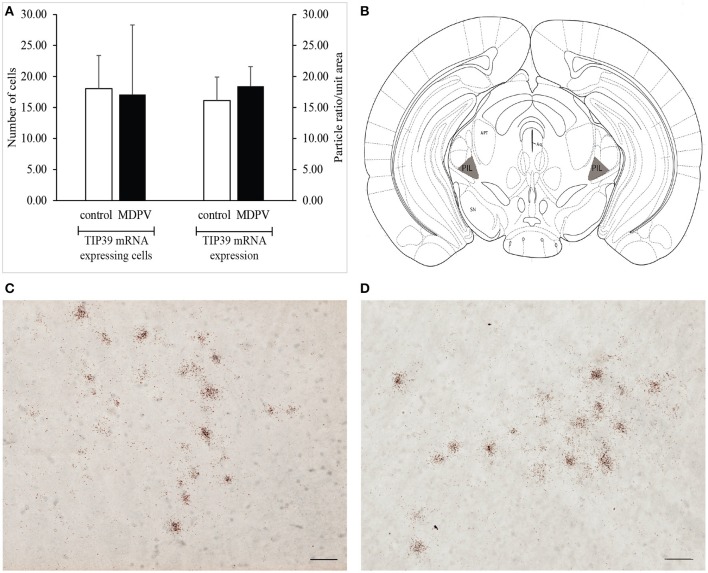
TIP39 mRNA expression in the posterior intralaminar complex of the thalamus (PIL) as revealed by *in situ* hybridization using antisense [^35^S]UTP-labeled riboprobes. **(A)** The values represent the total number of labeled cells identified within the reference space per hemisphere (left panel), as well as the density of silver grains marking the presence of mRNA transcript (right panel). We found no difference in the number of TIP39 mRNA expressing cells between the treatment groups. The values are expressed as the mean + S.E.M. **(B)** Localization of the PIL in a coronal section (gray shaded areas on diagram). Based on original template by Paxinos and Franklin (2008), at AP 0.10 mm rostral to the bregma. **(C)** Bright-field image of a representative autoradiogram displaying TIP39 mRNA expressing neurons in the PIL region of a control animal. **(D)** Representative autoradiogram depicting TIP39 mRNA expressing neurons in the MPO of an MDPV-treated animal. Scale bar: 30 μm **(C,D)**. Aq, cerebral aqueduct; APT, anterior pretectal nucleus; SN, substantia nigra. Counterstaining: Giemsa.

TIP39 mRNA positive perikarya could be detected in the PIL region of the brain specimens examined. Since there are no TIP39 mRNA positive cells present in the surrounding areas of the PIL, the area in focus was easy to demarcate. We did not find any significant difference either between the mean number of the TIP39 mRNA expressing neurons in the PIL, or between the TIP39 mRNA densities of the selected areas, respectively, in the two treatment groups (Student's *t*-test: *t* = 0.292 df = 5 *p* = 0.781, and *t* = −0.371 df = 5 *p* = 0.725) (Figure 9).

**Figure 10:**
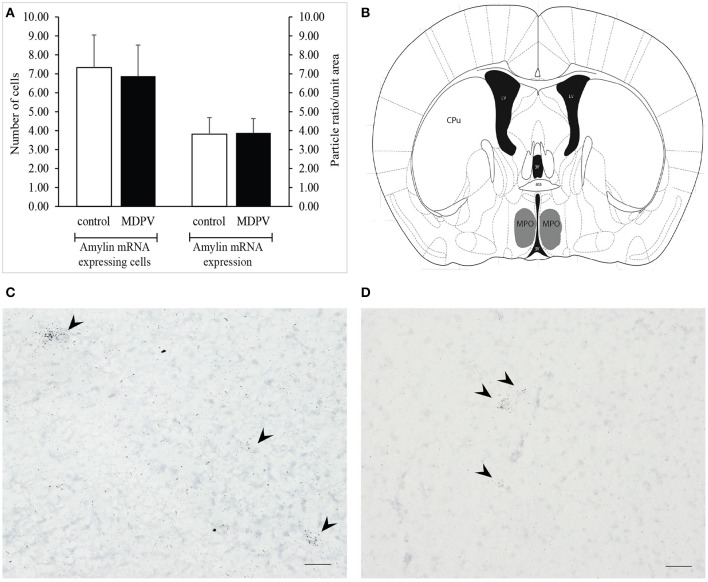
Amylin mRNA expression in the medial preoptic nucleus (MPO) as revealed by *in situ* hybridization using antisense [^35^S]UTP-labeled riboprobes. **(A)** The values represent the total number of labeled cells identified within the reference space per hemisphere (left panel), as well as the density of silver grains marking the presence of mRNA transcript (right panel). There was no significant difference in the number of amylin mRNA expressing cells between the treatment groups. The values are expressed as the mean + S.E.M. **(B)** Localization of the MPO in a coronal section (gray shaded area). Based on original template by Paxinos and Franklin (2008), at AP −3.08 mm from bregma. **(C)** Bright-field image of a representative autoradiogram displaying amylin mRNA expressing neurons in the MPO of a control animal. **(D)** Representative autoradiogram depicting amylin mRNA expressing neurons in the MPO of an MDPV-treated animal. Scale bar: 30 μm **(C,D)**. aca, anterior commissure, CPu, caudate putamen, LV, lateral ventricle, 3V, third ventricle. Counterstaining: Giemsa.

The cells expressing amylin mRNA were found in relatively low numbers in the medial preoptic nucleus (MPO). The cells were typically arranged in small clusters containing 2–5 cells and/or scattered throughout the MPO. Just like in case of the TIP39, we could not detect any significant difference between the total numbers of amylin mRNA expressing neurons or between the amylin mRNA densities of the cells in the two treatment groups (Student's *t*-test *t* = 0.254 *df* = 5 *p* = 0.809, and *t* = −0.108 *df* = 5 *p* = 0.917, respectively; Figure 10).

## Discussion

MDPV acts as a potent blocker of dopamine transporter and norepinephrine transporter, with moderate effect on serotonin uptake (Baumann et al., 2013; Simmler et al., 2013). Unlike other psychoactive cathinones (e.g., mephedrone, methylone), MDPV is not a transporter substrate (Reith et al., 2015; Glennon and Young, 2016). This mode of action is quite unique among synthetic cathinones, since methylone and mephedrone (other frequently used members of the family) are non-selective blockers of both dopamine-, norepinephrine- and serotonin-transporters (Cameron et al., 2013; Schindler et al., 2015). MDPV induces an outward current, similar to that elicited by cocaine, its effect being much more potent and lasting than that of cocaine (Cameron et al., 2013). Thus, the predominant action of MDPV is exerted via dopaminergic activation (Rickli et al., 2015; Shekar et al., 2017). This is further reflected in the profile of behavioral alterations following administration of MDPV and related drugs. Briefly, MDPV is a dose-dependent reinforcer in a continuous self-administration schedule (Watterson et al., 2014). In addition, it acts as a locomotor stimulant, bringing about stereotypies, also elevating heart rate, blood pressure and body temperature (Marusich et al., 2012; Baumann et al., 2013; Fantegrossi et al., 2013).

Chronic *in utero* treatment with MDPV led to no visible alteration in the resting behavior of the offspring. However, as revealed by motility-related tests, the drug exposed animals showed greater spontaneous motional activity, already at the age of 7 days after birth, while still lacking vision. Both the results of open field tests and those of force plate actometry point in the same direction, i.e., a wider occupancy of space available to the animal, whereas a genuine increase of the distance traveled, suggestive though, could not be verified statistically. The apparent discrepancy is likely due to a substantial vectorial contribution of non-ambulatory movements to the distance parameter (measured by actometry only). The control animals' non-locomotor movements of head, trunk and limbs may well compensate for a reduced locomotion of the animals. Taken together, increased motility of MDPV treated pups means more frequent and erratic deflections in multiple directions, rather than a longer course traveled in a given direction. Such motional profile likely represents increased motor excitation (agitation).

The results of the grip strength test do not support deterioration of motor coordination by the drug, at least in the case of young pups, since the MDPV treated animals performed equally well. However, this is not the case with chronically treated adults, whose latency scores were poorer than those of control animals, possibly due to an attention deficit.

Most dopamine promoting drugs, when administered to pregnant mothers, tend to impair motor functions in the newborn pups. Neurotoxic effects of prenatal MA exposure on serotonergic neurons are thought to be associated with learning impairment, behavioral deficits, increased motor activity and enhanced conditioned avoidance responses (for recent reviews see Ross et al., 2015; Smith et al., 2015). 3,4-Methylenedioxy-methamphetamine (MDMA), among other deleterious effects, diminished latency in wire hanging tests in mice (Kaizaki et al., 2014). MA, administered to pregnant female rats, was found to impair postural control of pups (PD 23) as detected by rotarod test and bar-holding test (Šlamberová et al., 2006; Pometlová et al., 2009). An early, but not late, exposure to d-methamphetamine during pregnancy markedly decreased early locomotion activity counts of rat pups (measured by photocell activity meter) (Acuff-Smith et al., 1996). Early exposure in this case corresponded to E7-E12, rather similar to the experimental protocol of the present study. Yet, in our case, an obvious impairment of motor coordination or locomotion was not observed, presumably due to an inherent difference between the action mechanisms of methamphetamine and cathinone families. In our case, the animals showed enhanced motility, possibly reflecting agitation and increased excitability, in agreement with previous observations in adult mice (Marusich et al., 2012; Fantegrossi et al., 2013). Hyperactivity and enhanced (non-discriminate) pecking was also described for young post-hatch domestic chicks under the effect of acute MDPV treatment, whereas other closely related compounds such as butylone suppressed motional activity (Zsedényi et al., 2014).

In our study, the time window for the MDPV injections corresponded to the second half of the first trimester in human development. Based on existing data, the drug was present in the pregnant female mouse at a time of the differentiation of dopaminergic (tyrosine hydroxylase expressing) neurons but prior to the appearance of D1 and D2 dopamine receptors (Wahlstrom et al., 2010). Thus, in so far as dopaminergic mechanisms are involved in the observed changes, they likely affected the presynaptic, rather than the postsynaptic side. As a potential mechanism, neurodegeneration (enhanced apoptosis) in striatum and nucleus accumbens has been demonstrated by our group in young postnatal mouse pups following systemic bolus injection of MDPV (Ádám et al., 2014). The issue of potential neurotoxicity and neurodegenerative effects of synthetic cathinones, including MDPV, is rather controversial (for a detailed review see: Angoa-Perez et al., 2017). MDPV and methylone do not affect striatal tyrosine hydroxylase (TH) or dopamine (DA) levels which would be considered as markers of neurotoxicity to monoaminergic nerve terminals in the striatum (Anneken et al., 2015; López-Arnau et al., 2015; Miner et al., 2017). Moreover, they do not cause elevation in striatal glial fibrillary acidic protein (GFAP) levels, pointing to an absence of astrogliosis in that area (Anneken et al., 2015). In addition, MDPV, methylone, and mephedrone fail to cause chronic depletion of DA, serotonin, or norepinephrine, which would be indicative of neurotoxicity (Angoa-Perez et al., 2012, 2014; Baumann et al., 2013). In a study on the monoamine receptor and transporter interaction profiles of various NPS, among others MDPV, no cytotoxicity was found even at the highest concentration, when tested in functional assays (Rickli et al., 2015). However, in other studies, cytotoxic effects of MDPV have been observed: in human DA-ergic SH-SY5Y cells MDPV caused apoptosis through the rise of reactive oxygen species (ROS), mitochondrial dysfunction and autophagy, which clearly shows the neurotoxic potential of MDPV, at least *in vitro* (Valente et al., 2017a,b). Overall, *in vitro* neurotoxicity of MDPV is possible, but more and more data suggest that this is unlikely to occur *in vivo*, with one important *caveat*. Almost all of the *in vivo* studies have used adult animals as model objects, however, our previous study showed that the neurotoxic potential might differ in adults and in pups (Ádám et al., 2014). Taking this fact and other CNS-related side effects, reported by clinicians, into consideration, the possibility of neurodegeneration and neurotoxicity underlying certain effects of MDPV should not be excluded (Blum et al., 2013).

Summarizing this part of the study, our results on pups clearly demonstrate that chronic systemic administration of the cathinone agent MDPV to female mice in a critical time period of pregnancy can exert *postpartum* effects on the offspring. Some of these effects (enhanced motional activity) may match those already reported for acute (bolus) injections of MDPV in young postnatal mice (Ádám et al., 2014).

As for the effect on dams, a significant reduction of maternal care was apparent in the drug treated mothers, most significantly in the latency of complete pup retrieval. As evidenced by the motility-related tests, neither the mothers nor their offspring had restricted agility to a degree which could hamper the exertion of pup retrieval behavior. Reduction in the quality of nests built by drug treated mothers can also be taken as a sign of maternal negligence, which, unlike pup retrieval, is not affected by the behavior of the offspring.

Concerning potential mechanisms of action, dopaminergic drugs are known to affect maternal behavior of rats. Severe deficits in pup retrieval have been reported after dopamine depletion in the striatum (Henschen et al., 2013). Chronic and acute haloperidol treatment disrupted pup retrieval and nest building (Li et al., 2004, 2005). Pre-treatment with the dopamine receptor D_2_-D_3_ (D2R/D3R) agonist quinpirole reversed these deficits (Zhao and Li, 2009) and the nucleus accumbens may be the specific site of drug action (Miller and Lonstein, 2005; Zhao and Li, 2010). In lactating rats, acute infusion of dopamine receptor D_1_ (D1R) antagonist SCH23390, but not the D2R antagonist eticlopride, into the nucleus accumbens disrupted pup retrieval (Numan et al., 2005). Following the dams' first experience with pups, sulpiride infusion into the nucleus accumbens delayed the re-onset of maternal behavior after 10 days of separation from pups (Parada et al., 2008).

As an alternative explanation, dysregulation of hypothalamic peptides (amylin, TIP39) was also considered. Amylin mRNA expressing cells are activated during the early postnatal period, as demonstrated by c-Fos immunochemistry and by increase in mRNA level (Szabó et al., 2012). TIP39 neurons of the PIL are activated in lactating mothers by suckling, whilst their position overlaps with the area of the afferent neuronal fibers underlying the suckling reflex (Cservenák et al., 2010). Another likely effect of TIP39 is reduced anxiety during lactation (LaBuda et al., 2004; Fegley et al., 2008). TIP39 neurons in the PIL markedly induce their mRNA transcript level as they convey suckling information toward the hypothalamus, thereby contributing to prolactin release from the pituitary and to the maintenance of maternal motivation (Cservenák et al., 2013). However, as evidenced by the present study, neither of these neuropeptides showed an altered level of expression in response to MDPV treatment. Thus, the findings do not support direct impairment of the hypothalamic regulation of maternal care. Therefore, the mechanism of action would be more in agreement with dopaminergic dysregulation, manifested in attention deficit, agitation, and negligence. Such alterations were observed also in the drug-elicited behavior of the pups.

Although the observed deficiencies of maternal behavior are likely due to negligence on the dam's side, possibly based on perturbation of dopaminergic mechanisms, alteration of the pups' behavior might also have accounted for the observed deterioration of maternal care. Some of the natural elicitors of maternal behavior, for example, ultrasonic calls (Ehret and Haack, 1984) may also have changed, as reported for chronic exposure to cocaine (Hess et al., 2002).

## Author contributions

LG: The Ph.D. student who played a role in performing all the steps of the work; AC: The head of the laboratory who helped in designing the experimental setup and writing the manuscript; GZ: Did the statistical analysis of the work; LG and LS: Invented and established the setup used for “force plate actometry,” and analyzed the data gained with it; ÁD: Helped in designing and executing the *in situ* hybridization experiment for TIP39 and amylin; ÁÁ: The principal investigator, who played a major role in designing and executing the experiment, and also in writing the manuscript.

### Conflict of interest statement

The authors declare that the research was conducted in the absence of any commercial or financial relationships that could be construed as a potential conflict of interest.
